# Medial Olivocochlear Reflex Strength Predicts Musical Perception and Speech‐in‐Noise Performance in Nonmusicians

**DOI:** 10.1002/brb3.71599

**Published:** 2026-07-14

**Authors:** İrem Sendesen, Suna Tokgöz Yılmaz

**Affiliations:** ^1^ Department of Audiology Gazi University Ankara Turkey; ^2^ Audiology Department Faculty of Health Science Ankara University Ankara Turkey

**Keywords:** auditory efferent system, medial olivocochlear reflex, musical perception, nonmusicians, otoacoustic emissions, speech‐in‐noise perception

## Abstract

**Objective:**

The medial olivocochlear (MOC) reflex enhances the signal‐to‐noise ratio (SNR) in the cochlea and is implicated in auditory scene analysis. Although musicians often exhibit robust MOC function, it remains unclear whether related auditory advantages stem from innate predisposition or training‐induced plasticity. This study investigated the relationship between individual variations in MOC reflex function, musical perception, and speech‐in‐noise (SIN) performance in nonmusicians.

**Methods:**

Fifty‐eight normal‐hearing nonmusicians were divided into two groups on the basis of the magnitude of their MOC reflex strength, quantified via contralateral suppression of transient evoked otoacoustic emissions (cTEOAEs) measured separately in each ear. Participants with suppression values above the sample median were assigned to the high MOC function group, whereas those with values below the median comprised the low MOC function group. All participants underwent pure‐tone and high‐frequency audiometry, auditory efferent system evaluation, speech‐in‐noise (SIN) testing at 0 and −10 dB SNRs, and musical perception assessment using the Montreal Battery for Evaluation of Amusia (MBEA).

**Results:**

The group with stronger MOC reflex activity demonstrated significantly better performance on MBEA scale and memory subtests and superior SIN perception at 0 dB SNR. Hierarchical regression confirmed MOC strength as a significant predictor of SIN performance at −10 dB SNR. Mediation analysis revealed that musical perception abilities partially mediated the relationship between MOC function and SIN performance.

**Conclusion:**

Variations in MOC reflex function among nonmusicians constitute a foundational biological trait that shapes auditory perception, supporting both musical abilities and speech‐in‐noise processing in the absence of formal musical training.

## Graphical abstract

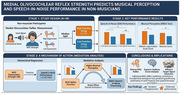

## 1 Introduction

The auditory efferent system, particularly the medial olivocochlear (MOC) reflex, operates as a brainstem‐mediated circuit that actively regulates cochlear input. By targeting the outer hair cells, the MOC system modulates their electromotility and reduces the gain of the cochlear amplifier (Guinan 2006; Lopez‐Poveda 2018). This physiological modulation can be assessed noninvasively in humans using the contralateral suppression of otoacoustic emissions (OAEs). As OAEs arise directly from outer hair cell electromotility, the reduction in their amplitude during contralateral noise stimulation provides a valid and widely used proxy for quantifying MOC reflex strength. This suppression effect functions as an “antimasking” mechanism that improves the signal‐to‐noise ratio (SNR) at the auditory periphery, a process indispensable for speech perception in noise (Lopez‐Poveda 2018; Perrot and Collet 2014). Furthermore, such efferent modulation is proposed to facilitate auditory stream segregation, enhancing listeners’ ability to extract relevant signals in complex acoustic environments.

Although superior auditory abilities and stronger MOC reflex responses are often observed in musicians, it remains unclear whether these characteristics stem from training‐induced plasticity or innate biological predisposition (Kraus and Chandrasekaran 2010; Perrot and Collet 2014). Furthermore, inconsistencies in the literature regarding MOC reflex strength suggest that methodological variations and the confounding effects of musical training complicate these findings (Stuart and Daughtrey 2016; Tarnowska et al. 2020). Importantly, MOC reflex strength is highly variable across different assessment metrics, suggesting that observed differences may largely reflect individual physiological diversity rather than musicianship alone. Consequently, determining the true functional impact of the efferent system requires examining individual differences in a population free from the influence of formal auditory training. By focusing on nonmusicians, it is possible to isolate baseline biological traits from experience‐dependent changes.

The role of the efferent system in speech‐in‐noise (SIN) perception is similarly subject to conflicting empirical findings. Although MOC‐mediated noise suppression theoretically facilitates SIN performance, some studies report positive links between auditory prowess and SIN perception (Bidelman and Bhagat 2015; Bidelman and Howell 2016), whereas others find no association (Mishra and Lutman 2014; Wagner et al. 2008). Crucially, studies involving trained listeners may obscure these relationships due to compensatory neural mechanisms. Therefore, investigating the interplay between MOC function, musical perception, and SIN performance in nonmusicians is essential. This approach allows for the assessment of whether natural variations in efferent inhibition serve as a foundational mechanism for robust auditory perception, independent of the cognitive and neural enhancements associated with musical expertise.

To address these gaps, this study investigates the interplay between musical perception, efferent function, and SIN performance in nonmusicians. On the basis of the “antimasking” role of the efferent system, we hypothesize that intrinsic MOC reflex strength serves as a direct predictor of psychoacoustic performance. We posit that by reducing cochlear gain and improving the SNR at the auditory periphery, a robust MOC reflex enhances the neural representation of complex acoustic features. Therefore, we expect individuals with stronger innate efferent suppression to exhibit superior outcomes in both musical perception tasks—which require precise spectral resolution—and speech‐in‐noise processing, even in the absence of formal musical training. In doing so, this study offers a novel framework to evaluate the functional impact of the MOC reflex on complex auditory processing.

## 2 Materials and Methods

### 2.1 Participants

This study included 58 volunteers aged between 19 and 30 years. Participants were recruited from a pool of individuals who applied to the Audiology Department for a routine hearing evaluation. Participation was strictly voluntary, and no financial compensation or academic credit was provided for their involvement in the study. The recruitment process followed a stepwise screening procedure. First, a comprehensive medical history was obtained, and otoscopic and tympanometric examinations were performed. Individuals with a history of otologic, neurologic, or psychiatric conditions, as well as those with abnormal middle ear findings or air–bone gaps exceeding 10 dB, were excluded. Subsequently, pure‐tone audiometry was conducted. Participants had normal‐hearing thresholds (≤20 dB HL) at all tested frequencies from 125 to 8000 Hz. Pure‐tone averages (PTA), calculated at 0.5, 1, 2, and 4 kHz, were also within normal limits for all participants.

Participants were divided into two groups on the basis of their global bilateral mean MOC suppression values (averaged across 1, 1.4, 2, 2.8, and 4 kHz for both ears). First, the amount of suppression was calculated for each ear individually by subtracting the overall TEOAE (transient evoked otoacoustic emission) response level in the presence of contralateral noise from the response level in quiet (Suppression (dB) = TEOAE(quiet) − TEOAE(noise)). To characterize the individual's systemic efferent tone and minimize the influence of ear‐specific variability, the arithmetic mean of the right and left ear suppression values was calculated for each participant. On the basis of this bilateral average, participants were categorized using a median‐split approach. This dichotomization was implemented primarily to establish distinct physiological profiles and facilitate clear group‐based comparisons (e.g., analyses of variance [ANOVAs]). However, to circumvent the loss of variance associated with dichotomization, the unadjusted, continuous MOC suppression values were strictly preserved for all predictive statistical modeling (i.e., hierarchical regression and mediation analyses). The sample's median suppression value was 1.16 dB. Participants with a bilateral average >1.16 dB were assigned to Group 1 (high MOC function), whereas those with an average <1.16 dB were assigned to Group 2 (low MOC function).

Participants also reported their daily average music listening duration (in hours) via a demographic questionnaire to assess informal auditory exposure. This study was conducted in accordance with the Declaration of Helsinki, and the study protocol was approved by the Ethics Committee of Ankara University (approval no.: 2023/271). The written consent of the participants was obtained. The study was carried out in two stages. In the first stage, participants underwent pure‐tone audiometry, high‐frequency audiometry, speech perception in noise, and auditory efferent system evaluation. In the second stage, musical perception was evaluated. The entire testing procedure took approximately 90 min to complete.

### 2.2 The First Stage

#### 2.2.1 Pure‐Tone Audiometry and High‐Frequency Audiometry Test

All audiological evaluations and experimental procedures were conducted in a double‐walled, sound‐treated booth meeting ANSI standards to minimize ambient noise levels. Standard pure‐tone audiometry (125–8000 Hz) was conducted using an Interacoustics AC40 clinical audiometer with TDH‐39P headphones. For extended high‐frequency audiometry (9–18 kHz), the same audiometer was used with Sennheiser HDA 200 circumaural headphones, which are calibrated for high‐frequency testing. Hearing thresholds were assessed across the frequency range of 125–8000 Hz. Only individuals with air‐conduction thresholds ≤20 dB HL at all tested frequencies were included in the study (Clark 1981).

In the high‐frequency audiometry test, thresholds were measured using pure‐tone stimuli at 9, 10, 12, 14, 16, and 18 kHz (Tonndorf and Kurman 1984). The hearing thresholds obtained during this test were documented on standardized data collection forms for evaluation at the end of the study.

#### 2.2.2 Auditory Efferent System Evaluation

The auditory efferent system was assessed using the contralateral suppression of transient evoked otoacoustic emissions (cTEOAEs) test. The assessment was performed via a computer‐based application using earphones. All measurements were carried out with the Otodynamics ILO 292 USB II system. During testing, continuous broadband white noise at approximately 60 dB SPL—comparable to conversational speech levels—was delivered to one ear. This intensity level was carefully calibrated to ensure that it did not trigger the middle ear reflex. Simultaneously, a linear click stimulus at an intensity of 60 dB peSPL (peak‐equivalent sound pressure level) was presented to the opposite ear through an earphone equipped with an in‐ear microphone. Linear clicks were deliberately chosen over nonlinear clicks, as they are essential for accurately capturing the efferent suppression effect without the interference of artifact‐cancellation algorithms. To ensure high‐quality recordings, response acceptance criteria were applied: Only emissions demonstrating a minimum wave reproducibility of 70% and a SNR of at least 3 dB at the analyzed frequency bands were included in the analysis. This allowed for real‐time measurement of the auditory response and reliable evaluation of contralateral suppression. The test was terminated after 260 sweeps were completed (approximately 1040 clicks) and was conducted separately for the right and left ears. TEOAE responses were recorded across the 1000–4000 Hz frequency range under two conditions: in quiet and during contralateral noise presentation (Moulin et al. 1993).

#### 2.2.3 Speech Perception in Noise

Speech perception in noise was assessed using a calibrated AC40 clinical audiometer with TDH‐39 headphones. Speech stimuli consisted of phonetically balanced word lists, each containing 25 monosyllabic words. The test was conducted under four experimental conditions: right ear at 0 dB SNR, right ear at −10 dB SNR, left ear at 0 dB SNR, and left ear at −10 dB SNR. To accommodate these conditions, a total of four distinct but psychometrically equivalent lists were utilized (one for each condition). To prevent learning, order, or fatigue effects, both the sequence of the test conditions (e.g., testing ear and SNR level) and the assignment of specific word lists to those conditions were fully counterbalanced and randomized across all participants.

Speech was presented at each participant's most comfortable listening level (MCL) to ensure audibility and comfort, thereby reducing performance variability due to differences in loudness perception and minimizing potential confounding effects of presentation level on SIN performance, a practice supported by standard clinical and research protocols (Killion and Niquette 2000; Wilson 2011). The MCL was established individually for each ear prior to testing using a standard clinical ascending‐descending method with continuous running speech. Participants were instructed to verbally indicate when the speech volume was comfortably clear—neither too soft to require straining nor loud enough to cause discomfort. White noise served as the competing stimulus and was delivered at 50 dB SPL, a level comparable to conversational speech. The use of white noise, while spectrally less speech‐like than multi‐talker babble, provides a consistent, spectrally broad masker that challenges the auditory system's ability to extract signals based on spectral and temporal fine structure cues, which are precisely the cues the MOC reflex is theorized to protect (Bidelman and Bhagat 2015; Lopez‐Poveda 2018). This choice allows for a more direct assessment of the peripheral efferent system's contribution to signal‐in‐noise extraction without the additional cognitive‐linguistic load associated with informational masking from competing speech. The test was conducted under two SNR conditions: 0 and −10 dB. Speech perception was evaluated separately for the right and left ear conditions. During the right ear condition, speech stimuli were delivered to the right ear, whereas noise was presented to the left ear; the reverse was applied for the left ear condition.

This contralateral noise presentation paradigm was deliberately chosen to parallel the contralateral suppression paradigm used in the cTEOAE assessment of MOC reflex function. In both procedures, the signal of interest (speech or click stimuli) is presented to the test ear while competing noise is presented to the opposite ear, thereby engaging the crossed MOC pathway. This methodological alignment allows for a more direct comparison between efferent system function and SIN performance, as both tasks assess the capacity of the MOC reflex to extract signals in the presence of contralateral interference (Kumar et al. 2016). Participants were instructed to repeat each word they heard, and performance was quantified as the percentage of correctly identified words.

### 2.3 The Second Stage

#### 2.3.1 Montreal Battery of Evaluation of Amusia (MBEA)

In the second stage, musical perception was assessed using the MBEA, a computer‐based test with established validity and reliability (Vuvan et al. 2018). Although originally developed to diagnose congenital Amusia, the MBEA has been widely established in the literature as a sensitive instrument for quantifying individual differences in musical perception abilities among the general population, including nonmusicians (Peretz et al. 2003; Swaminathan and Schellenberg 2018). To evaluate the core domains of musical processing while minimizing participant fatigue within the comprehensive test protocol, three representative subtests were strategically selected: the scale test (assessing pitch organization), the meter test (assessing temporal organization), and the memory test. This selection allows for a focused assessment of the distinct neural subsystems involved in music processing (melodic vs. temporal) without the redundancy of the full battery. Each subtest consisted of 30 pairs of melodies presented through headphones at the participant's MCL.

In the scale test, one melody in each pair contained a critical pitch alteration, specifically, a change in the pitch of one tone to a note outside the established tonality (key violation) while preserving the original melodic contour. Participants were asked to indicate whether the two melodies in each pair were the same or different.

In the meter test, participants were presented with two harmonized melodies and were instructed to identify which melody was in a duple meter (march) and which was in a triple meter (waltz).

In the final stage, the memory test assessed musical memory by presenting participants with a total of 30 melodies: 15 previously heard during the scale and meter tests, and 15 novel melodies. Participants were asked to indicate whether they recognized each melody as familiar or unfamiliar.

Prior to each subtest, participants completed a short practice trial consisting of two sample melodies that were not included in the analysis. An answer sheet was provided for all subtests, and participants were instructed to mark their responses for each item.

Performance on the MBEA was quantified as the percentage of correct responses, calculated separately for the scale, meter, and memory subtests.

### 2.4 Statistical Analysis

All statistical analyses were conducted using R version 4.3.1 (R Foundation for Statistical Computing, Vienna, Austria). The alpha level was set at 0.05 for all statistical tests, and all tests were two‐tailed. To comprehensively evaluate the role of MOC reflex strength, a dual analytical strategy was employed. First, primary analyses (ANOVAs) utilized a categorical approach (median split) to establish distinct physiological profiles (high vs. low MOC) and facilitate intuitive group‐level comparisons. Second, advanced statistical modeling (hierarchical regression and mediation) utilized the unadjusted, continuous MOC suppression values to preserve the full spectrum of data variance, thereby accurately quantifying predictive relationships. This complementary approach ensures both clinical interpretability and statistical robustness, providing convergent validity for the findings. Data were screened for normality using Shapiro–Wilk tests and visual inspection of *Q*–*Q* plots, with no substantial violations detected. Homogeneity of variance was assessed using Levene's test. Where violations occurred (specifically, for comparisons between the high and low MOC function groups), appropriate corrections were applied using Welch's ANOVA for unequal variances.

#### 2.4.1 Primary Analyses

Group comparisons for continuous variables were conducted using independent samples *t*‐tests, with Welch's correction applied when homogeneity of variance assumptions were violated. Categorical variables were analyzed using chi‐square tests of independence, with Fisher's exact test employed when expected cell frequencies were below 5. Effect sizes for *t*‐tests are reported as Cohen's *d*, interpreted as small (0.20), medium (0.50), and large (0.80). For chi‐square tests, phi coefficients (for 2 × 2 tables) and Cramer's *V* (for larger contingency tables) are reported, interpreted as small (0.10), medium (0.30), and large (0.50).

Mixed‐design ANOVAs were employed for repeated measures data, including the 2 (group) × 7 (frequency) × 2 (ear) ANOVA for extended high‐frequency thresholds and the 2 (group) × 5 (frequency) × 2 (ear) ANOVA for MOC suppression values. The 2 (group) × 2 (SNR) × 2 (ear) mixed ANOVA was used for speech‐in‐noise performance. Mauchly's test of sphericity was conducted for within‐subjects factors, and Greenhouse–Geisser corrections were applied when sphericity assumptions were violated. Partial eta‐squared (*η*_p_^2^) values are reported as measures of effect size, with values of 0.01, 0.06, and 0.14 representing small, medium, and large effects, respectively.

Multivariate analysis of variance (MANOVA) was conducted to examine group differences across the three MBEA subtests (scale, meter, and memory) simultaneously, with Wilks’ lambda used as the test statistic. Follow‐up univariate ANOVAs were conducted for significant multivariate effects.

#### 2.4.2 Advanced Statistical Modeling

Hierarchical multiple regression analysis was performed to predict speech‐in‐noise performance at −10 dB SNR. Demographic variables (age, daily music listening hours) were entered in Step 1, with bilateral MOC activity (AvrSnrBil) added in Step 2. Model assumptions, including linearity, homoscedasticity, independence of errors, and absence of multicollinearity, were verified through residual analysis and variance inflation factors (all VIFs < 2.0).

Mediation analysis was conducted using the lavaan package in R to examine whether musical perception (MBEA scale scores) mediated the relationship between efferent function (AvrSnrBil) and speech‐in‐noise performance. The analysis employed maximum likelihood estimation with 5000 bootstrap samples to generate bias‐corrected confidence intervals for the indirect effect, a robust method recommended for assessing indirect effects in smaller samples (Hayes 2017). Model fit was assessed using multiple indices, including comparative fit index (CFI > 0.95) and root mean square error of approximation (RMSEA < 0.08).

Cluster analysis was performed using *k*‐means clustering (Hartigan and Wong 1979) with *k* = 3 to identify distinct auditory profiles on the basis of efferent function, speech‐in‐noise performance, and musical perception abilities. Variables were standardized prior to analysis to ensure equal contribution to distance calculations, and the optimal number of clusters was determined using the elbow method and silhouette analysis. The resulting cluster solution was validated through discriminant function analysis and cross‐tabulation with experimental groups.

#### 2.4.3 Post Hoc Analyses and Multiple Comparisons

For significant main effects and interactions in ANOVA models, post hoc comparisons were conducted using Tukey's HSD test to control for Type I error inflation. Effect sizes for post hoc comparisons are reported as mean differences with 95% confidence intervals. For chi‐square analyses with significant overall effects, standardized residuals were examined to identify specific cells contributing to the significance.

All statistical outputs are reported in accordance with American Psychological Association (APA) guidelines, including exact *p* values (unless *p* < 0.001), effect sizes, and confidence intervals where appropriate.

## 3 Results

### 3.1 Participant Characteristics and Group Equivalence

A total of 58 participants were included in the analysis (Group 1: *n* = 29, Group 2: *n* = 29). Table 1 presents demographic characteristics and baseline measures for both groups. Independent samples *t*‐tests revealed no significant differences between groups in age (*t*(56) = 0.31, *p* = 0.759), daily music listening hours (*t*(56) = 0.62, *p* = 0.541), or instrumental training duration (*t*(56) = 1.37, *p* = 0.177), confirming successful group matching on demographic variables. Although duration of dance training was recorded to account for potential sensorimotor effects on rhythm perception, no significant differences were found between groups, *t*(56) = 1.01, *p* = 0.43, and this variable did not correlate with musical perception or speech‐in‐noise performance.

**TABLE 1:** Demographic characteristics, music background, and baseline measures by group.

Variable	Group 1 (*n* = 29)	Group 2 (*n* = 29)	*t*/*χ* ^2^	*p*	Cohen's *d*/*φ*
**Demographic characteristics**					
Age (years)	22.73 ± 4.78	22.25 ± 3.78	*t*(56) = 0.42	0.74	0.11
Gender (women/men)	25/4	24/5	*χ* ^2^(1) = 0.12	0.87	0.05
**Music background**					
Daily music listening (hours)	1.76 ± 0.84	2.93 ± 2.78	*t*(56) = −2.17	0.10	−0.57
Duration of instrument training (months)	1.47 ± 3.67	20.52 ± 49.08	*t*(56) = −2.08	0.10	−0.55
Duration of dance training (months)	2.88 ± 2.54	2.28 ± 1.95	*t*(56) = 1.01	0.43	0.27
**Preferred music genres**	*n* (%)	*n* (%)	*χ* ^2^(4) = 6.84	0.16	0.34
Pop	13 (44.8)	9 (31.0)			
Rock	0 (0.0)	3 (10.3)			
Slow	5 (17.2)	5 (17.2)			
Rap	2 (6.9)	1 (3.4)			
Classical	3 (10.3)	0 (0.0)			
**Auditory measures**					
Extended high‐frequency average (dB HL)	12.34 ± 6.78	11.89 ± 7.21	*t*(56) = 0.25	0.68	0.07

*Note*: *N* = 58 (*n* = 29 per group). Group 1 = low MOC activity (AvrSnrBil ≤ 1.16 dB); Group 2 = high MOC activity (AvrSnrBil > 1.16 dB). Values represent mean ± standard deviation for continuous variables and frequency (percentage) for categorical variables. Independent samples *t*‐tests were used for continuous demographic and auditory comparisons. Pearson's chi‐square tests (*χ* ^2^) were utilized for categorical variables. Degrees of freedom are reported in parentheses. Cohen's *d* effect sizes are interpreted as small (0.20), medium (0.50), and large (0.80). No statistically significant differences were found between groups on any demographic or baseline auditory variable (all *p* > 0.05), ensuring successful group matching prior to experimental testing.

### 3.2 Hearing Thresholds

**FIGURE 1:**
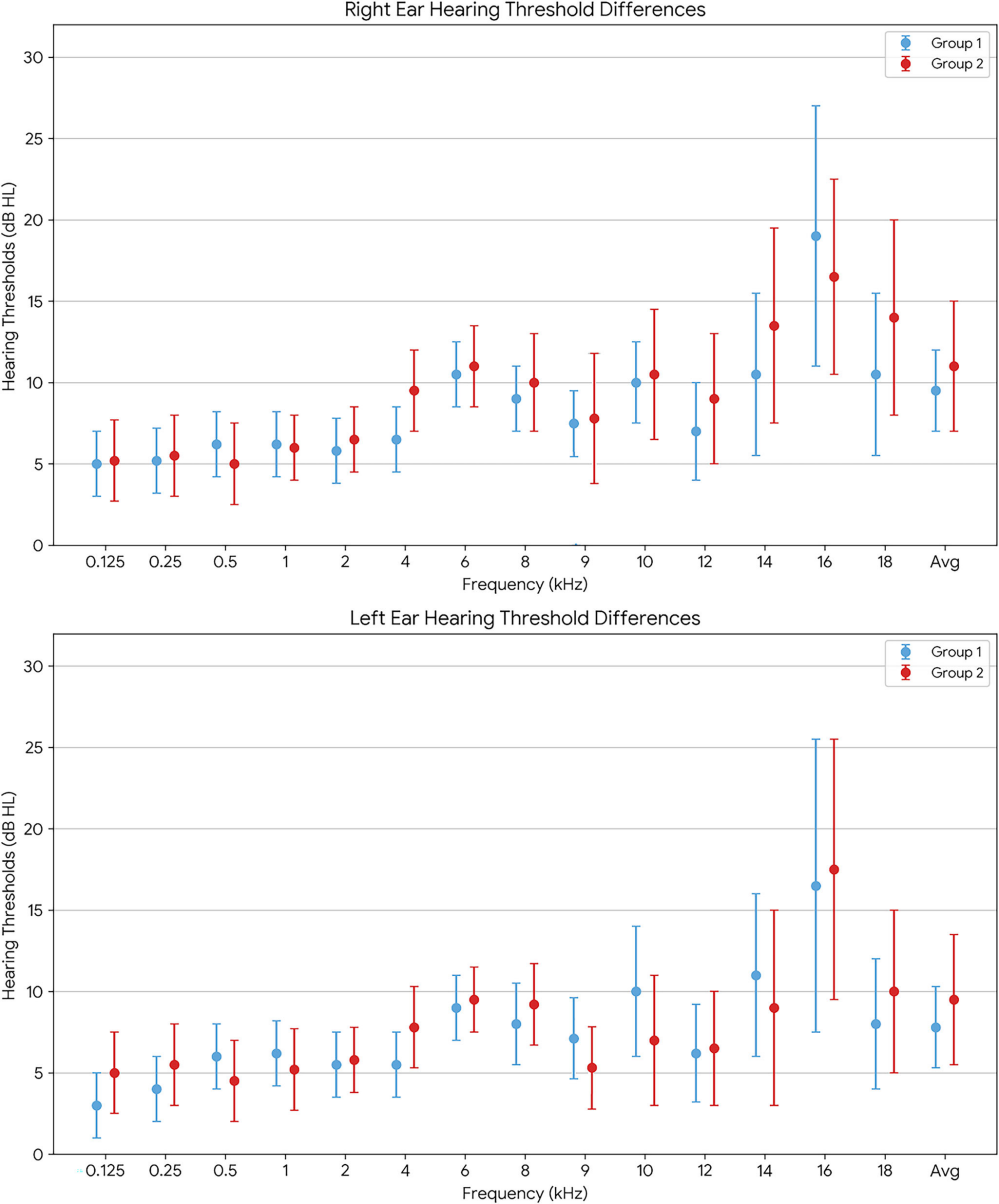
Comparisons of hearing thresholds (0.125–18 kHz) between groups. The figure displays hearing thresholds across standard and extended high frequencies (0.125–18 kHz). The top panel illustrates right ear thresholds, and the bottom panel illustrates left ear thresholds. Circles represent mean thresholds (dB HL) for Group 1 (low MOC function, blue, *n* = 29) and Group 2 (high MOC function, red, *n* = 29). Error bars indicate standard deviations (±1 SD). “Avg” refers to the arithmetic mean of thresholds across the measured extended high‐frequency range (9–18 kHz). A 2 (group) × 6 (frequency) × 2 (ear) mixed‐design ANOVA revealed no significant group × frequency interaction (*F*(6, 336) = 1.23, *p* = 0.289), indicating comparable extended high‐frequency hearing sensitivity between the nonmusician groups. Total sample size *N* = 58.

A 2 (group) × 6 (frequency: 9–18 kHz) × 2 (ear: right/left) mixed‐design ANOVA on hearing thresholds revealed significant main effects of frequency (*F*(5, 336) = 18.92, *p* < 0.001, *η*_p_^2^ = 0.25) and ear (*F*(1, 56) = 6.45, *p* = 0.014, *η*_p_^2^ = 0.10), with better thresholds in the right ear overall. However, no significant group × frequency interaction was found (*F*(6, 336) = 1.23, *p* = 0.289), indicating comparable extended high‐frequency hearing sensitivity between groups across all tested frequencies. Hearing thresholds are shown in Figure 1.

### 3.3 Auditory Efferent System Function

**FIGURE 2:**
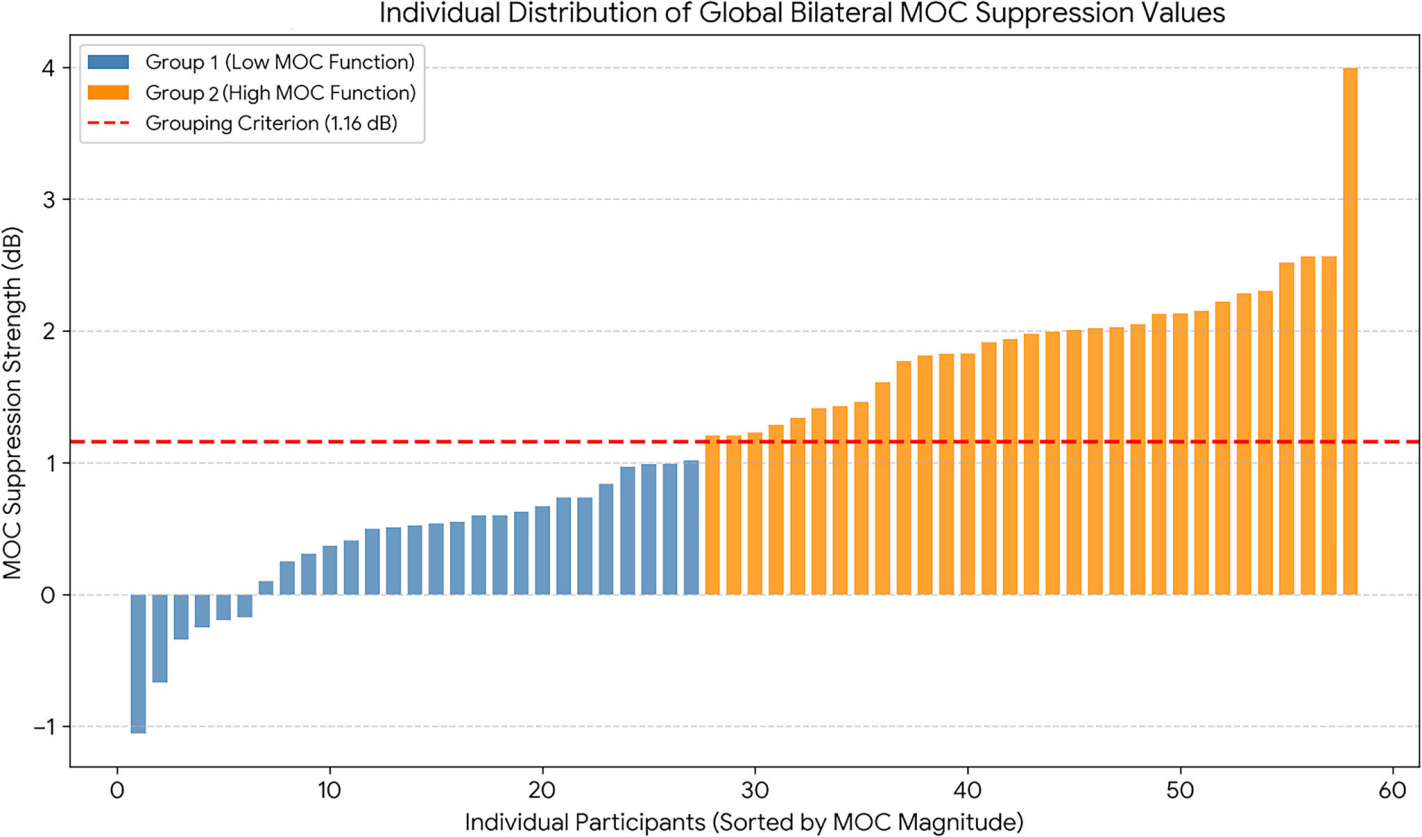
Individual distribution of global bilateral MOC reflex suppression strength. Bars represent individual participants (*N* = 58) sorted by their global bilateral MOC suppression magnitude. The red dashed line indicates the median split criterion (1.16 dB) used for group assignment. Participants with continuous values above this threshold were assigned to Group 2 (high MOC function, orange bars, *n* = 29), whereas those with values below were assigned to Group 1 (low MOC function, blue bars, *n* = 29). MOC, medial olivocochlear.

Table 2 summarizes MOC suppression values across frequencies and ears. A 2 (group) × 5 (frequency: 1–4 kHz) × 2 (ear) mixed ANOVA revealed a significant main effect of group (*F*(1, 56) = 52.37, *p* < 0.001, *η*_p_^2^ = 0.48). It should be explicitly noted that this robust group difference is an expected outcome stemming directly from our median‐split grouping criterion (Figure 2), which was specifically utilized to isolate distinct efferent profiles (a methodological aspect further elaborated in Section 4). The analysis also revealed significant main effects for both frequency (*F*(4, 224) = 9.83, *p* < 0.001, *η*_p_^2^ = 0.15) and ear (*F*(1, 56) = 5.12, *p* = 0.028, *η*_p_^2^ = 0.08). Integrating these overall effects with our descriptive statistics, we observed that MOC suppression was significantly stronger overall in the right ear (2.22 ± 0.84 dB) compared to the left ear (1.80 ± 0.92 dB). Crucially, these main effects were qualified by a significant group × frequency interaction (*F*(4, 224) = 3.45, *p* = 0.009, *η*_p_^2^ = 0.06). Post hoc Tukey HSD analyses demonstrated that although the high‐MOC group (Group 2) exhibited stronger suppression across the entire spectrum, this physiological advantage was most pronounced at the 2 and 2.8 kHz frequency bands (both *p* < 0.001). For instance, aligning with our descriptive data at 2 kHz in the right ear, Group 2 exhibited a robust mean suppression of 4.78 ± 2.45 dB, whereas Group 1 demonstrated only 1.45 ± 1.89 dB. Furthermore, post hoc analysis of the frequency main effect confirmed that, across all participants, efferent suppression peaked at 2 kHz, which was significantly greater than the suppression observed at 1.4 kHz (*p* = 0.003) and 4 kHz (*p* = 0.015).

**TABLE 2:** Medial olivocochlear (MOC) suppression values by frequency, ear, and group.

Frequency (kHz)	Ear	Group 1	Group 2	*F*	*p*	*η*_p_^2^
1	Right	1.23 ± 1.45	3.87 ± 2.12	28.92	<0.001	0.34
1	Left	0.89 ± 1.67	4.12 ± 2.34	35.67	<0.001	0.39
1.4	Right	−0.67 ± 2.12	2.45 ± 2.78	24.56	<0.001	0.31
1.4	Left	−1.23 ± 2.45	1.89 ± 2.67	22.34	<0.001	0.29
2	Right	1.45 ± 1.89	4.78 ± 2.45	38.92	<0.001	0.41
2	Left	0.78 ± 2.01	4.23 ± 2.56	34.78	<0.001	0.38
2.8	Right	0.89 ± 1.78	3.67 ± 2.34	31.45	<0.001	0.36
2.8	Left	0.45 ± 1.92	3.12 ± 2.45	26.78	<0.001	0.32
4	Right	1.67 ± 1.56	2.89 ± 2.12	6.89	0.011	0.11
4	Left	1.34 ± 1.67	2.45 ± 2.23	4.78	0.033	0.08

*Note: N* = 58 (*n* = 29 per group). Values represent mean suppression (dB) ± standard deviation. Positive values indicate contralateral suppression of transient evoked otoacoustic emissions (TEOAEs), whereas negative values indicate enhancement. A 2 (group) × 5 (frequency) × 2 (ear) mixed‐design ANOVA was performed. The analysis revealed significant main effects of group (*F*(1, 56) = 52.37, *p *< 0.001), frequency (*F*(4, 224) = 9.83, *p *< 0.001), and ear (*F*(1, 56) = 5.12, *p* = 0.028), as well as a significant group × frequency interaction (*p* = 0.009). Post hoc Tukey HSD tests indicated that Group 2 showed significantly stronger suppression, particularly at 2 and 2.8 kHz (both *p *< 0.001). Partial eta‐squared (*η*_p_^2^) represents effect size. Grouping was performed on the basis of the global bilateral mean; thus, values at specific frequencies may individually exceed the 1.16 dB global cutoff due to frequency‐band variability.

### 3.4 Speech‐in‐Noise Perception

Table 3 displays speech recognition scores under different SNR conditions. A 2 (group) × 2 (SNR: 0 dB/−10 dB) × 2 (ear) mixed ANOVA on speech recognition scores revealed significant main effects of group (*F*(1, 56) = 15.28, *p* < 0.001, *η*_p_^2^ = 0.21), SNR (*F*(1, 56) = 187.42, *p* < 0.001, *η*_p_^2^ = 0.77), and ear (*F*(1, 56) = 8.93, *p* = 0.004, *η*_p_^2^ = 0.14). Crucially, a significant group × SNR interaction was observed (*F*(1, 56) = 9.65, *p* = 0.003, *η*_p_^2^ = 0.15), indicating that Group 2 maintained better performance, particularly under the more challenging −10 dB SNR condition. Post hoc simple effects analysis of the group × SNR interaction confirmed that although both groups performed worse at −10 dB SNR compared to 0 dB SNR (both *p* < 0.001), the performance decrement from 0 to −10 dB SNR was significantly smaller in Group 2 (mean difference = −21.8%) compared to Group 1 (mean difference = −26.9%), *F*(1, 56) = 9.65, *p* = 0.003. Furthermore, Group 2 significantly outperformed Group 1 at both the 0 dB SNR (*p* = 0.008) and −10 dB SNR (*p* < 0.001) conditions.

**TABLE 3:** Speech recognition scores (%) by signal‐to‐noise ratio (SNR) condition, ear, and group.

SNR condition (dB)	Ear	Group 1	Group 2	*F*	*p*	*η*_p_^2^
0	Right	82.4 ± 10.2	89.3 ± 8.1	8.34	0.006	0.13
0	Left	80.1 ± 11.3	87.2 ± 9.4	7.23	0.010	0.11
−10	Right	56.7 ± 12.8	68.9 ± 11.2	15.67	<0.001	0.22
−10	Left	54.2 ± 13.5	66.3 ± 12.1	13.45	0.001	0.19

*Note: N* = 58 (*n* = 29 per group). Values represent mean percent correct speech recognition scores (%) ± standard deviation. A 2 (group) × 2 (SNR) × 2 (ear) mixed ANOVA was conducted, revealing significant main effects of group (*F*(1, 56) = 15.28, *p* < 0.001), SNR (*F*(1, 56) = 187.42, *p* < 0.001), and ear (*F*(1, 56) = 8.93, *p* = 0.004). A significant group × SNR interaction (*F*(1, 56) = 9.65, *p* = 0.003) was also observed. Post hoc simple effects analysis confirmed that Group 2 maintained significantly better speech‐in‐noise performance, particularly under the more challenging −10 dB SNR condition (*p* < 0.001). Partial eta‐squared *η*_p_^2^) represents effect size.

### 3.5 Musical Perception Abilities

Table 4 presents MBEA subtest scores. A MANOVA on the three MBEA subtests (scale, meter, and memory) revealed a significant multivariate effect of group (Wilks’ *λ* = 0.79, *F*(3, 54) = 4.82, *p* = 0.005). Follow‐up univariate ANOVAs showed that Group 2 outperformed Group 1 on the scale subtest (*F*(1, 56) = 12.47, *p* = 0.001, *η*_p_^2^ = 0.18) and memory subtest (*F*(1, 56) = 6.32, *p* = 0.015, *η*_p_^2^ = 0.10), but not on the meter subtest (*F*(1, 56) = 2.18, *p* = 0.145). Post hoc comparisons using Tukey's HSD confirmed the significant pairwise differences between groups for the scale (*p* = 0.001) and memory (*p* = 0.015) subtests.

**TABLE 4:** Montreal Battery of Evaluation of Amusia (MBEA) subtest scores by group.

Subtest	Group 1	Group 2	*F*(1, 56)	*p*	*η*_p_^2^
Scale	75.3 ± 9.2	81.4 ± 7.1	12.47	0.001	0.18
Meter	66.8 ± 11.5	70.2 ± 10.3	2.18	0.145	0.04
Memory	73.6 ± 8.9	78.9 ± 7.4	6.32	0.015	0.10

*Note: N* = 58 (*n* = 29 per group). Values represent mean percent correct responses ± standard deviation. A multivariate analysis of variance (MANOVA) revealed a significant overall multivariate effect of group (Wilks’ *λ* = 0.79, *F*(3, 54) = 4.82, *p* = 0.005). Follow‐up univariate ANOVAs showed that Group 2 outperformed Group 1 specifically on the scale subtest (*p* = 0.001) and memory subtest (*p* = 0.015), but not on the meter subtest (*p* = 0.145).

### 3.6 Hierarchical Multiple Regression Analysis

Table 5 summarizes the hierarchical regression analysis predicting SIN performance at −10 dB SNR. To examine the predictive value of efferent function on auditory performance, a hierarchical multiple regression was conducted with SIN performance at −10 dB SNR as the dependent variable. Demographic variables (age, music listening hours) were entered in Step 1, explaining 8% of the variance (*R^2^* = 0.08, *F*(2, 55) = 2.39, *p* = 0.102). Although instrumental training duration was also considered as a potential covariate, it was excluded from the final model as independent samples *t*‐tests showed no significant differences between groups (*p* = 0.177), and its inclusion did not significantly improve the variance explained in Step 1. Addition of AvrSnrBil in Step 2 significantly improved the model (Δ*R* ^2^ = 0.24, *F*(1, 54) = 18.73, *p* < 0.001), with the final model explaining 32% of the variance in SIN performance (*F*(3, 54) = 8.52, *p* < 0.001). AvrSnrBil emerged as the strongest predictor (*β* = 0.52, *p* < 0.001). Examination of the regression coefficients in the final model indicated that for every 1 dB increase in bilateral MOC suppression (AvrSnrBil), SIN performance at −10 dB SNR improved by approximately 3.2 percentage points (*B* = 3.19, SE = 0.74, *p* < 0.001), holding age and music listening constant.

**TABLE 5:** Hierarchical regression predicting SIN performance at −10 dB signal‐to‐noise ratio (SNR).

Predictor	*β*	*t *	*p *	Δ*R* ^2^	*F* change
Step 1				0.08	2.39
Age	−0.18	−1.34	0.186		
Music listening	0.12	0.89	0.378		
Step 2				0.24	18.73
Age	−0.15	−1.28	0.206		
Music listening	0.09	0.76	0.451		
AvrSnrBil	0.52	4.33	<0.001		
Total *R* ^2^	0.32*				

*Note: N* = 58. SIN = speech‐in‐noise; AvrSnrBil = continuous global bilateral MOC suppression. Dependent variable: SIN performance at −10 dB SNR. *β* = standardized regression coefficient. A two‐step hierarchical multiple linear regression was conducted. Demographic variables (age, daily music listening hours) were entered in Step 1, explaining 8% of the variance (ns). The addition of continuous MOC function (AvrSnrBil) in Step 2 significantly improved the model (Δ*R* ^2^ = 0.24, *p* < 0.001). The final full model significantly predicted 32% of the variance in SIN performance (*F*(3, 54) = 8.52, *p* < 0.001), demonstrating that intrinsic MOC reflex strength is a robust predictor of auditory performance in noise.

*p < 0.001.

The analysis specifically focused on the −10 dB SNR condition, as the antimasking benefits of the MOC reflex are most critical and observable under challenging listening environments with high background noise, whereas 0 dB SNR may not fully tax the efferent system.

To determine the unique predictive value of efferent function, variables were entered in two steps. In Step 1, age and daily music listening hours were entered as control variables to account for basic demographic factors and informal musical exposure. In Step 2, the global bilateral mean MOC suppression (AvrSnrBil) was added to evaluate its unique contribution to SIN performance above and beyond the covariates.

### 3.7 Mediation Analysis

Table 6 presents the mediation analysis results examining whether musical perception mediated the relationship between efferent function and SIN performance. A mediation analysis using 5000 bootstrap samples examined whether musical perception (MBEA scale scores) mediated the relationship between efferent function (AvrSnrBil) and SIN performance. The indirect effect was significant (*β* = 0.18, 95% CI [0.06, 0.33]), indicating partial mediation, with 42% of the total effect of efferent function on SIN performance operating through enhanced musical perception. The model demonstrated good fit to the data (CFI = 0.98, RMSEA = 0.05, SRMR = 0.04). The path from AvrSnrBil to MBEA scale scores was significant (*a* = 0.31, *p* = 0.001), as was the path from MBEA scale scores to SIN performance (*b* = 0.29, *p* = 0.008). The direct effect (*c*′) of AvrSnrBil on SIN performance remained significant after accounting for the mediator (*c*′ = 0.34, *p* < 0.001), confirming partial mediation.

**TABLE 6:** Mediation analysis of efferent function on SIN performance through musical perception.

Effect	Estimate	SE	95% CI	*p*
Direct effect (*c*)	0.34	0.08	[0.18, 0.50]	<0.001
Indirect effect (*a* × *b*)	0.18	0.07	[0.06, 0.33]	0.008
Total effect (*c* + *a* × *b*)	0.52	0.09	[0.34, 0.70]	<0.001

*Note: N* = 58. SIN = speech‐in‐noise. The table presents the results of a mediation analysis conducted with maximum likelihood estimation using 5000 bootstrap samples to examine whether musical perception (MBEA scale scores) mediated the relationship between continuous efferent function (AvrSnrBil) and SIN performance. The indirect effect was statistically significant (*β* = 0.18, *p* = 0.008, 95% CI [0.06, 0.33]), indicating partial mediation. Specifically, 34.6% of the total effect of efferent function on SIN performance operates through enhanced musical pitch perception abilities.

### 3.8 Cluster Analysis of Auditory Profiles

Table 7 shows the distribution of participants across the three identified auditory profiles and their association with experimental groups. A *k*‐means cluster analysis (*k* = 3) based on efferent function (AvrSnrBil), SIN performance (at −10 dB SNR), and musical perception revealed three distinct auditory profiles. Given that the meter subtest did not show significant group differences, musical perception was operationalized specifically using the MBEA scale subtest scores, ensuring that the clustering algorithm was driven by the variables demonstrating the most robust physiological and perceptual variance. This analysis yielded: (1) “high performers” (*n* = 21) with strong efferent function and superior auditory performance; (2) “moderate performers” (*n* = 25); and (3) “low performers” (*n* = 12) with compromised efferent and perceptual abilities. Chi‐square analysis confirmed a significant association between cluster membership and experimental group (*χ* ^2^(2) = 15.38, *p* < 0.001). Post hoc examination of standardized residuals revealed that Group 2 (high MOC) was significantly overrepresented in the “high performers” cluster (standardized residual = 3.1) and underrepresented in the “low performers” cluster (standardized residual = −2.1). Conversely, Group 1 (low MOC) was significantly overrepresented in the “low performers” cluster (standardized residual = 2.1) and underrepresented in the “high performers” cluster (standardized residual = −3.1).

**TABLE 7:** Distribution of participants across auditory profiles by experimental group.

Auditory profile	Group 1	Group 2	Total
High performers	4	17	21
Moderate performers	16	9	25
Low performers	9	3	12
**Total**	**29**	**29**	**58**

*Note: N* = 58 (*n* = 29 per group). Values represent the number of participants (*n*) and their corresponding row percentages. Auditory profiles were identified using a *k*‐means cluster analysis (*k* = 3) on the basis of unadjusted continuous variables (MOC suppression, −10 dB SIN performance, and MBEA scale scores). A Pearson's chi‐square test of independence confirmed a significant association between cluster membership and the experimentally assigned median‐split group (*χ* ^2^(2) = 15.38, *p* < 0.001). Group 2 (high MOC) is significantly overrepresented in the “high performers” profile.

## 4 Discussion

The present study provides compelling evidence that individual differences in MOC reflex function, as quantified by cTEOAEs, are significantly associated with auditory perceptual abilities in individuals without formal musical training, accounting for a substantial portion of the variance in speech‐in‐noise performance. Our central finding is that nonmusicians with stronger MOC reflex activity demonstrated superior performance compared to their counterparts with weaker MOC function in both musical perception tasks—specifically melodic structure discrimination and musical memory—and speech‐in‐noise (SIN) perception, particularly under the 0 dB SNR condition. This pattern of results directly implicates the MOC reflex in auditory scene analysis—the perceptual process of segregating and grouping acoustic components to form distinct auditory objects. The stronger ability of the high‐MOC group to extract melodic contours from complex musical sequences and to understand speech in competing noise reflects improved signal‐background segregation, a core operation of auditory scene analysis. By refining cochlear gain and improving the neural representation of target signals in noise, the efferent system facilitates the listener's capacity to parse complex auditory scenes into relevant figure‐ground relationships. These findings underscore the foundational role of the auditory efferent system in refining cochlear output to support complex auditory scene analysis, independent of experience‐driven neural plasticity associated with long‐term musical practice (Kraus and Chandrasekaran 2010; Perrot and Collet 2014).

The observed right‐ear advantage for SIN perception in the high‐efferent group offers an intriguing clue regarding the underlying neural circuitry. This finding aligns with neuroimaging evidence linking successful SIN perception to left‐hemisphere dominance in auditory processing (Bidelman and Howell 2016). Given the predominantly contralateral anatomical projection of the MOC pathways (Perrot and Collet 2014), stronger right‐sided MOC projections to the left cochlea could theoretically enhance the neural representation of speech signals arriving via the right ear (left hemisphere), thereby facilitating its analysis in the language‐dominant hemisphere. This interpretation finds resonance in bihemispheric models of speech comprehension, which posit that although bilateral fronto‐temporal networks support fundamental auditory competition, the left inferior frontal cortex is specialized for decomposing complex linguistic inputs (Bozic et al. 2010).

A pivotal finding from our advanced statistical modeling is that the relationship between robust MOC function and enhanced SIN performance is partially mediated by superior musical perception abilities, specifically sensitivity to melodic contour. This mediation effect suggests a shared processing mechanism, whereby a more finely tuned efferent system enhances the spectral resolution of acoustic inputs, which, in turn, benefits both the extraction of pitch patterns in music and the segregation of speech formants from a noisy background. This finding provides a mechanistic link, suggesting that the perceptual advantages often attributed to musical training may, in part, be predicated on pre‐existing, innate differences in low‐level efferent circuitry (Kraus and Chandrasekaran 2010; Strait et al. 2012).

The context‐dependent nature of MOC function was starkly revealed by the differential results across SNR conditions. Although the high‐efferent group excelled at 0 dB SNR, their advantage dissipated at the more challenging −10 dB SNR. This dissociation indicates that the benefits conferred by a robust peripheral efferent system have functional limits; under conditions of extreme acoustic degradation, the antimasking capabilities of the MOC reflex are likely overwhelmed (Acuña et al. 2023). This result corroborates the findings of Wagner et al. (2008), who reported no correlation between SIN intelligibility and efferent activity in normal‐hearing individuals, and suggests that in such demanding listening situations, central cognitive processes—such as auditory working memory and attentional control—usurp the primary role in perception (Parbery‐Clark et al. 2011; Strait et al. 2012).

Furthermore, the differential impact of MOC strength across musical subtests is highly informative. The significant group differences on the scale (melodic) and memory subtests, but not on the meter (rhythmic) subtest, imply that the MOC reflex is more critically involved in the spectral fine‐structure analysis required for pitch and key processing than in the temporal envelope processing required for meter discrimination. This dissociation aligns with models that attribute auditory working memory and complex pattern recognition to cortical and hippocampal systems, which operate somewhat independently of the peripheral efferent gain control (Zatorre et al. 2007). The absence of correlations between MOC strength and pure‐tone thresholds or TEOAE amplitudes reinforces the concept that the MOC reflex functions as a dynamic efferent gain control system, specialized for optimizing SNRs in challenging listening environments, rather than determining absolute auditory sensitivity (Lopez‐Poveda 2018). This dissociation suggests that the efferent system constitutes a distinct functional component of auditory processing, operating independently of the mechanisms that govern detection thresholds in quiet.

It is also important to acknowledge the potential confounding role of informal musical background within our cohort. Although there were no statistically significant differences between the groups regarding daily music listening hours and duration of instrumental training (*p* = 0.10), the moderate effect sizes (*d* = −0.57 and *d* = −0.55, respectively) indicate that the high‐MOC group had a trend toward slightly greater informal exposure to music. Given that even informal musical engagement can subtly refine auditory processing and speech‐in‐noise abilities, this difference warrants consideration as a potential confounding factor. However, our advanced statistical modeling was structured specifically to account for this variance. The hierarchical regression model demonstrated that when basic demographic factors and informal musical exposure were controlled for in Step 1, they did not significantly predict SIN performance (explaining only 8% of the variance, *p* = 0.102). Therefore, although informal musical background may exert a mild supportive influence, the robust predictive power of MOC suppression (*β* = 0.52, *p* < 0.001) clearly emerges as the primary physiological driver of the observed perceptual advantages.

Methodologically, our use of cTEOAE suppression across multiple frequencies provided a sensitive measure of efferent function, consistent with protocols known to robustly capture MOC‐induced effects in the mid‐frequency range where the reflex is most potent (Kumar et al. 2016; Moulin et al. 1993). Furthermore, our group assignment based on the sample median cTEOAE value provided a clear, data‐driven method to compare individuals with relatively higher and lower innate efferent function, effectively addressing our research aim. The methodological choices for SIN testing—using MCL and white noise—were deliberate. Testing at MCL controlled for loudness growth differences, while using white noise allowed us to specifically probe the efferent system's ability to extract signals in a challenging energetic masking environment, isolating its contribution from the effects of informational masking (Bidelman and Bhagat 2015; Lopez‐Poveda 2018).

In conclusion, this study suggests innate MOC reflex function as a key biological trait that shapes an individual's aptitude for both musical and speech perception in noise. Our findings delineate a neurobiological pathway wherein superior efferent control enhances spectral resolution, thereby delineating higher‐order auditory and cognitive processes. These results not only help resolve longstanding discrepancies in the literature regarding the relationship between musicianship and efferent function but also provide a foundational framework for future research. Subsequent investigations should employ longitudinal training designs and neuroimaging techniques to elucidate the causal interactions between innate predispositions and experience‐dependent plasticity. Extending this line of inquiry to clinical populations with known efferent deficits, such as children with auditory processing disorders (Muchnik et al. 2004), holds significant promise for developing targeted auditory training and rehabilitative interventions aimed at improving perceptual outcomes in challenging listening environments.

## Author Contributions

**Suna Tokgöz Yılmaz**: supervision, writing – review and editing, conceptualization, methodology. **İrem Sendesen**: conceptualization, methodology, writing – original draft, formal analysis, data curation.

## Funding

The authors have nothing to report.

## Ethics Statement

This study was conducted in accordance with the Declaration of Helsinki. The study protocol was approved by the Ethics Committee of Ankara University (approval no.: 2023/271).

## Consent

Written informed consent was obtained from all participants involved in the study prior to the evaluation.

## Conflicts of Interest

The authors declare no conflicts of interest.

## Data Availability

The data that support the findings of this study are available from the corresponding author upon reasonable request.
